# Impact of Citric Acid on the Structure, Barrier, and Tensile Properties of Esterified/Cross-Linked Potato Peel-Based Films and Coatings

**DOI:** 10.3390/polym16243506

**Published:** 2024-12-17

**Authors:** Katharina Miller, Corina L. Reichert, Markus Schmid, Myriam Loeffler

**Affiliations:** 1 Meat Technology & Science of Protein-Rich Foods (MTSP), Department of Microbial and Molecular Systems, Leuven Food Science and Nutrition Research Centre, KU Leuven Campus Ghent, B-9000 Ghent, Belgium; katharina.miller@kuleuven.be; 2 Sustainable Packaging Institute (SPI), Faculty of Life Sciences, Albstadt-Sigmaringen University, 72488 Sigmaringen, Germany; reichert@hs-albsig.de

**Keywords:** side stream valorization, biopolymers, barrier properties, chemical modification, food packaging application

## Abstract

The valorization of potato peel side streams for food packaging applications, especially for the substitution of current petrochemical-based oxygen barrier solutions such as EVOH, is becoming increasingly important. Therefore, potato peel-based films and coatings (on PLA) were developed containing 10–50% (*w*/*w* potato peel) citric acid (CA). To determine the impact of CA concentration on the structure and physicochemical properties of cast films and coatings, ATR-FTIR spectroscopy, moisture adsorption isotherms, tensile properties, light transmittance, oxygen permeability, carbon dioxide transmission rate, and water vapor transmission rate measurements were performed. The results indicate that an increase in CA concentration from 10% to 30% increased esterification/cross-linking and resulted in minimal values for the oxygen permeability (0.08 cm^3^ m^−2^ d^−1^ bar^−1^) at 50% RH and water vapor transmission rate (1.6 g m^−2^ d^−1^) at 50% → 0% RH, whereas an increase from 30% to 50% increased free CA concentration and resulted in increased flexibility, indicating that CA functioned as a plasticizer within the film/coating at higher concentrations. Overall, potato peel-based coatings containing CA showed comparable barrier properties to EVOH. We assume that an extensive industrial purification or fractionation of potato peel, which was not carried out in this study, could lead to even lower transmission rates.

## 1. Introduction

Over the past decade, research has explored the valorization of side streams from the potato processing industry for packaging applications by investigating the effects of various physical and chemical treatments on the physicochemical properties of potato peel-based films [1]. In the literature, high-pressure homogenization [2], ultrasonic treatment [3], and mild acid hydrolysis [4] have been found to induce favorable effects on several packaging-related properties of potato peel-based cast films, including film-forming ability, permeability, and mechanical properties. Previous studies demonstrated that cast film properties depend on the type and amount of plasticizer used [4,5]. In general, potato peel-based films plasticized with glycerol or sorbitol showed oxygen transmission rates comparable to those of polyamide films [5]. However, these potato peel-based films still have significant shortcomings compared to petrochemical-based plastics such as ethylene vinyl alcohol (EVOH), including higher permeability and processing challenges. The same limitations apply to more purified biopolymer films, such as those derived from starch and proteins [6]. Achieving properties comparable to EVOH, especially using an unpurified and alternative resource like potato peel, could make potato peel-based biopolymers a viable option as a barrier coating or layer in bio-based, circular food packaging. Therefore, further optimization strategies of the biopolymer’s properties are required [7,8].

Exploring various physical, chemical, and biochemical modification approaches for potato constituents in biopolymer packaging applications to address challenges such as water sensitivity has identified citric acid (CA) cross-linking as a promising solution [8]. In general, CA has been successfully used to cross-link starch, other carbohydrates, proteins, and various biopolymer blends through either solvent-based applications [9] or thermal processing [10], resulting in reduced water sensitivity, permeability, and migration as well as enhanced tensile properties [11,12,13]. Cross-links within starch and other carbohydrate polymers primarily occur in the form of di-ester linkages between their hydroxyl groups and the carboxylic groups of CA [14,15]. Within protein films, cross-links tend to occur through nucleophilic substitution between the partially negatively charged amine groups of proteins and the partially positively charged carboxylic groups of CA [12,16]. In addition, cross-linking can occur between the functional groups of amino acid side chains in proteins, such as di-sulfide bonds [17], or between the hydroxyl groups of the plasticizer and the carboxylic groups of CA [18]. However, when the concentration of CA exceeds a certain threshold, the cross-linking sites of the biopolymer become saturated, and the excess CA functions as a non-toxic plasticizer within the biopolymer matrix [19,20]. As a result, water sensitivity, permeation, and ductility increase while the tensile strength of the biopolymer decreases [9]. The influence of increasing CA concentration on the different physical properties, however, can vary greatly depending on the type of biopolymer and the plasticizer concentrations used [11,12,14].

Since low molecular weight substances such as sorbitol or glycerol have proven to be effective plasticizers in biopolymer films [21], most studies investigating CA cross-linking have used a base concentration of glycerol (15–33%), functioning as the film’s main plasticizer [9,22,23,24]. Using 15% glycerol as a plasticizer, the addition of CA (5–20%) resulted in decreased water sensitivity (moisture content, water solubility, and swelling degree), increased tensile properties (tensile strength and elongation at break), and reduced water vapor permeability in chitosan/starch films, due to the formation of cross-links [25]. A similar effect was observed in mung bean starch-based films containing 30% glycerol and 1–5% CA. However, the addition of 15% CA resulted in decreased mechanical properties compared to the control films [9]. Likewise, the addition of 10–30% CA to glycerol (30%)-plasticized hemicellulose films led to increased flexibility and a decrease in tensile strength, water solubility, and water vapor permeability [26]. The authors attributed this effect to “flexible cross-linking”, as CA incorporation simultaneously exhibited cross-linking and plasticizing effects [26].

Using CA without the addition of an additional plasticizer in the range of 5–30% within starch-based films, Menzel et al. [27] reported a decrease in water-soluble starch content and an increase in the degree of di-esterification while Olsson et al. [28] observed decreasing moisture content and diffusion coefficients with increasing CA concentration. Similarly, tensile strength increased, while ductility, water sorption, oxygen permeability, and water vapor transmission rate decreased with increasing CA concentration (5–15%) in horse gram protein films [29], indicating enhanced cross-linking. However, the plasticizing effect of CA was evident in sodium alginate [30] and PVA/xylan composite films [31], where tensile strength decreased, and elongation at break increased with increasing CA concentration (0–100% *w*/*w* alginate and 0–30% *w*/*w* PVA/xylan, respectively).

Based on the complex composition of potato peel, which includes starch, other carbohydrates, and proteins, various functional sites are available that could participate in cross-linking reactions. Given the dual role of CA as both a cross-linking agent and a plasticizer, this study aimed to determine the CA concentration at which its plasticizing effect outweighs its cross-linking function. Consequently, CA was used without the addition of an extra plasticizer at concentrations ranging from 10 to 50% (*w*/*w* potato peel), and the influence of CA concentration on the structure, tensile properties, and humidity-dependent barrier properties of potato peel-based films and coatings was investigated.

To our knowledge, this is the first study to report on a potato peel film-forming suspension being coated onto another substrate. The application of potato peel as a thin barrier coating/layer of <10 µm on PLA shifts the potential use of potato peel-based films/coatings from edible/antimicrobial coatings for food [32,33] or as standalone biodegradable films [34] towards functioning as an oxygen barrier in bi-layer or even multilayer systems, enabling the preservation of a variety of different food products. As PLA is an emerging biopolymer with increasing industrial applications in different sectors (biomedical, automotive, etc.) but exhibits insufficient barrier properties for most food packaging applications [35], the functionalization of PLA with a potato peel-barrier coating for food packaging is a promising approach considered in this study. Humidity-dependent oxygen permeability measurements were conducted for the first time using potato peel-based coatings. The results were linked to the corresponding moisture absorption isotherms to evaluate the material’s relative humidity-dependent behavior. Furthermore, ATR-FTIR analysis was performed on both the cationic and anionic forms of the carboxyl group within potato peel-based films. This analysis helped to partially elucidate the structure (the distinction between esterified and free CA) of potato peel-based films containing 0–50% (*w*/*w* potato peel) CA in order to support the physicochemical results of this study.

## 2. Materials and Methods

### 2.1. Materials

Citric acid (CA; purity ≥ 99.5%) was purchased from Carl Roth GmbH + Co. KG (Karlsruhe, Germany). Lithium chloride (purity ≥ 99%), potassium acetate (purity ≥ 99%), magnesium chloride hexahydrate (purity ≥ 99%), potassium carbonate (purity ≥ 99%), potassium chloride (purity ≥ 99%), and potassium nitrate (purity ≥ 99%) were purchased from Merck KGaA (Darmstadt, Germany). Magnesium nitrate (purity ≥ 98%), and hydrochloric acid (1 M) were purchased from Fisher Scientific (Kandel, Germany). Sodium chloride (purity ≥ 99%) and sodium nitrate (purity ≥ 99%) were purchased from VWR Chemicals (Leuven, Belgium). Sodium hydroxide (2 mol/L) was purchased from Häberle Labortechnik GmbH + Co. KG (Ettlenschieß, Germany). 

Deionized water was produced in-house at Albstadt-Sigmaringen University. A roll of 25 ± 2 µm thick PLA was provided by SÜDPACK Holding GmbH (Erlenmoos, Germany).

### 2.2. Development and Properties of Potato Peel Powder

Potato peel powder with an average particle size of D[4,3] = 27 µm was prepared from potato peel side streams (Bernina variety) that were kindly provided by Sautter Kartoffelverarbeitung (Bondorf, Germany). In brief, the freshly generated potato peel side stream was frozen at −18 ± 1 °C for storage purposes and then thawed at 3 ± 1 °C for 23 ± 1 h and dried at 50 °C for 32 h (UF 260, Memmert GmbH + Co. KG, Schwabach, Germany) in 10 kg batches. To obtain a fine powder, the dried material was grounded in two steps using a knife mill (GM 200, Retch GmbH, Haan, Germany) and a rotor mill (ZM 200, Retch GmbH, Haan, Germany). The composition of the potato peel powder was 81.9 ± 0.8% carbohydrates (thereof 62.4 ± 6.1% starch), 6.1 ± 0.2% proteins, 4.0 ± 0.0% ash, 0.7 ± 0.1% fat, and 7.4 ± 0.4% moisture content (Normec Servaco Food Control NV, Wetteren, Belgium). The potato peel powder was packed into aluminum bags and stored vacuum-sealed at room temperature until further use.

### 2.3. Preparation of the Film-Forming Suspension

First, 7% (*w*/*w*) potato peel suspensions were prepared in 1 L batches. The suspensions were pre-mixed with a spoon before being homogenized (200 rpm, 15 min) and heated (80 °C, 300 rpm, 30 min) using an electric stirrer (TM6, Vorwerk Elektrowerke GmbH & Co. KG, Wuppertal, Germany). After the suspension was cooled down to room temperature in an ice bath, 10–50% (*w*/*w* potato peel) CA was added, and the suspension was homogenized again at 200 rpm for 15 min using the same electric stirrer. Prior to the coating application and cast film preparation, air bubbles on the surface of the suspension were removed using a pipette. The pH values of the film-forming suspensions containing 10%, 30%, or 50% (*w*/*w* potato peel) CA were approximately 3.6, 3.1, and 2.3, respectively (FiveEasy Plus, Mettler Toledo, Gießen, Germany).

To investigate the influence of pH on the resulting properties of potato peel-based cast films and coatings, a supplementary study was conducted. In this study, the pH of suspensions containing 30% (*w*/*w*) CA was adjusted from 2.8 ± 0.2 to pH levels of 2, 3, 4, 5, and 6 using 1M HCl and 2M NaOH, respectively.

### 2.4. Cast Film Preparation

Cast films with a target thickness of approximately 150 µm were produced by pouring 18.7, 16.6, or 15.1 g of film-forming suspension containing 10%, 30%, or 50% CA (*w*/*w* potato peel), respectively, into 12 × 12 cm polystyrol petri dishes (Greiner Bio- One GmbH, Frickenhausen, Germany). To obtain comparable cross-linking of the cast films and coating, cast films were dried at 70 °C for 90 min in a forced convection dryer (UF 260, Memmert GmbH + Co. KG, Schwabach, Germany) and then placed into a climate chamber (KMF 720 Binder GmbH, Tuttlingen, Germany) for one week, enabling equilibration of the cast films at 23 °C and 50% relative humidity (RH), before films were peeled from the Petri dishes. Coherent cast films containing 0% CA (*w*/*w* potato peel) could not be obtained due to the cracking of the film upon film formation.

### 2.5. Cast Film Thickness

The thickness of cast films was determined according to Miller et al. [5] at five random positions by mechanical touch using a digital micrometer (EFU040, Autoutlet, Eformation Technology Limited, Guangzhou, China).

### 2.6. Coating Application

During the coating application, the film-forming suspension was constantly stirred using a magnetic stirrer (RCT 5 digital, IKA, Staufen, Germany). Coating of 25 ± 2 µm thick PLA sheets (A4) was performed using a laboratory coating unit (Coating unit CUF 5, Sumet Technologies GmbH & Co. KG, Denklingen, Germany). Approximately 4 mL of film-forming suspension was applied onto the substrate using a pipette and distributed by an 80 µm wire rod (80 µm wet thickness) across the PLA sheet at a speed of 1.2 m/min. The coating was dried at 70 °C for 10 min within the coating unit.

### 2.7. Coating Thickness

**Figure 1:**
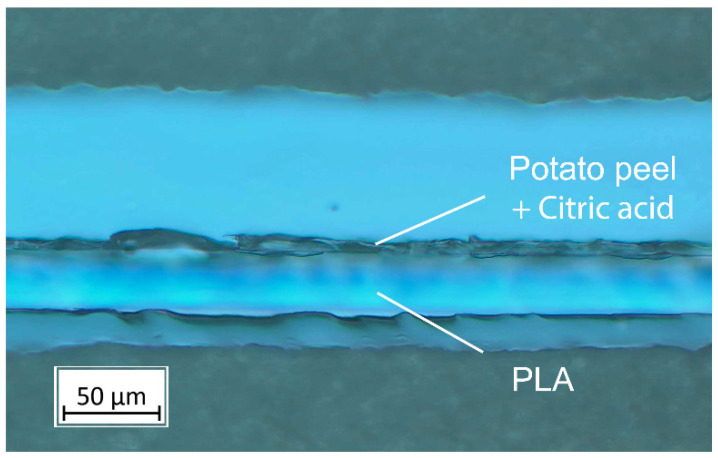
Cross-section of a PLA–potato peel bi-layer obtained by light microscopy.

The coating thickness was calculated from the dry mass of the wet coating thickness (80 µm × (0.07 + 0.077 or 0.091 or 0.105)) for 10%, 30%, or 50% CA, which is equivalent to 6.2, 7.3, and 8.4 µm, respectively. To check the dried thickness of the potato peel-based coatings, five random microtome cuts (Rotationsmikrotom CUT 4055, microTec Laborgeräte GmbH, Walldorf, Germany) from different sheets were taken and the thickness was examined microscopically (Zeiss Axioscope 5 & Axiocam 208c, Carl Zeiss Microscopy GmbH, Jena, Germany), as shown in Figure 1. Taking standard deviations from microscopic measurements, an average thickness of 7 ± 1 µm was assumed for all coated samples.

### 2.8. ATR-FTIR-Spectroscopy

Attenuated Total Reflection Fourier-Transform Infrared (ATR-FTIR) spectra of the cast films were recorded between 4000 and 600 cm^−1^ in threefold, using a macro module equipped with a diamond ATR-Crystal (Alpha II, Bruker Optik GmbH, Ettlingen, Germany). For each spectrum, 128 scans at 4 cm^−1^ resolution were performed after a background measurement was carried out. As carboxylic and ester groups of free and esterified CA are known to overlap in an IR spectrum, samples were also immersed according to Cuardo et al. [18] and Yang [36] for 2 min at room temperature in 0.1 M NaOH to induce a shift of carboxylic groups from approx. 1728 cm^−1^ to its carboxylate anion form at approx. 1589 cm^−1^ before being measured by ATR-FTIR as described above.

### 2.9. Moisture Adsorption Isotherms

Moisture adsorption measurements were performed by placing specimens (6 × 6 cm) into a desiccator cabinet (Nalgene Company, Rochester, NY, USA) containing different saturated salt solutions of known water activity. Specimens were stored within the desiccator until equilibrium was reached (weight increase < 0.001 g) but at least for one week, respectively. For each sample, a fourfold determination was carried out. First, samples were stored over silica gel to obtain a RH of 0%. Then, LiCl, CH_3_COOK, Mg(Cl)_2_, K_2_CO_3_, Mg(NO_3_)_2_, NaNO_3_, NaCl, KCl, and KNO_3_ were used to obtain a RH of 12%, 23%, 33%, 43%, 53%, 63%, 75%, 85%, and 93% at 23 ± 1 °C, respectively. Temperature and RH within the cabinets were controlled using thermohygrometers (Exacto, TFA-Dostmann, Wertheim, Germany). At the end of the experiment, specimens were dried at 50 °C for 72 h to determine the dry weight.

### 2.10. Tensile Properties

Tensile strength and elongation at break were determined according to DIN 527-3 [37]. Tensile measurements were performed using a tensile tester (C-Frame 5000 N, Alluris GmbH & Co. KG, Freiburg, Germany) equipped with a 50 N load cell. Equilibrated specimens (23 °C, 50% RH) of 15 mm width were tested in-between 50 mm-distanced grips at a test speed of 50 mm/min. Mean values were calculated from a tenfold determination that was carried out per sample.

### 2.11. Light Transmittance

The light transmittance of 7 ± 1 µm potato peel-based coatings on 25 ± 2 µm PLA sheets was determined in 10 nm steps between 360 nm and 740 nm using a spectrophotometer (CM-36dG, Konica Minolta Business Solutions Deutschland GmbH, Langenhagen, Germany). The transmittance spectrum of 25 ± 2 µm PLA sheets was used as a reference. For each sample, a fivefold determination was carried out.

### 2.12. Oxygen Permeability

The oxygen permeability (OP) of the samples was measured at 23 °C and 50–90% RH by the oxygen-specific carrier gas method [38] using an Oxtran 2/12 R (MOCON Inc., Minneapolis, MN, USA). For each sample, a twofold determination was performed. OP was measured using the convergence by cycle method with a maximum deviation of 2%. The individual zero of each cell was subtracted from the previously measured OP value (cm^3^ m^−2^ d^−1^ bar^−1^) at the end of the measurement. In addition, oxygen transmission rates (OTRs) measured at 23 °C and 50% RH were determined fourfold for statistical comparison.

### 2.13. Carbon Dioxide Transmission Rate

The carbon dioxide transmission rate (CO_2_TR) of the coated samples was measured at 23 °C and 50% RH, with the coating being exposed to the humid side, by the differential pressure method [39] using a gas transmission tester (GTT, Brugger Feinmechanik GmbH, Munich, Germany). For each sample, a threefold determination was performed. CO_2_TR was measured after an evacuation time of 1 h.

### 2.14. Water Vapor Transmission Rate

Water vapor transmission rate (WVTR) was determined using the gravimetric method [40] with silica gel-filled cups and a testing area of 50 cm^2^. For each sample, a fivefold determination was conducted. WVTR measurements were first conducted at 50% → 0% RH, followed by a second measurement at 85% → 0% RH, both performed in a climate chamber (KMF 720 Binder GmbH, Tuttlingen, Germany). For both measurements, the coating was exposed to the humid side.

### 2.15. Statistical Analysis

All statistical analyses were carried out using the software Minitab (Version 20.4, Minitab GmbH, Munich, Germany). Measured data (n ≥ 3) from tensile tests, OTR, CO_2_TR, and WVTR measurements were tested for outliers and normal distribution by the Dixon test (*p* < 0.05) and multiple probability plots (*p* < 0.05), respectively. Normal distributed data were tested for similar variances according to the Levene test (*p* < 0.05). Based on the result of the Levene test, an ANOVA according to Tuckey (same variances) or Games–Howell (different variances) was performed to identify significant differences (*p* < 0.05). Significant differences between samples are shown by the use of different superscript letters.

## 3. Results and Discussion

To examine the influence of CA concentration on the structure and physicochemical properties of potato peel-based coatings/films, cast films were characterized by ATR-FTIR (Section 3.1), moisture adsorption isotherms (Section 3.2), and tensile testing (Section 3.3). Coatings were evaluated by light transmittance (Section 3.4) and humidity-dependent barrier measurements against oxygen, carbon dioxide, and water vapor (Section 3.5).

### 3.1. ATR-FTIR Spectroscopy

**Figure 2:**
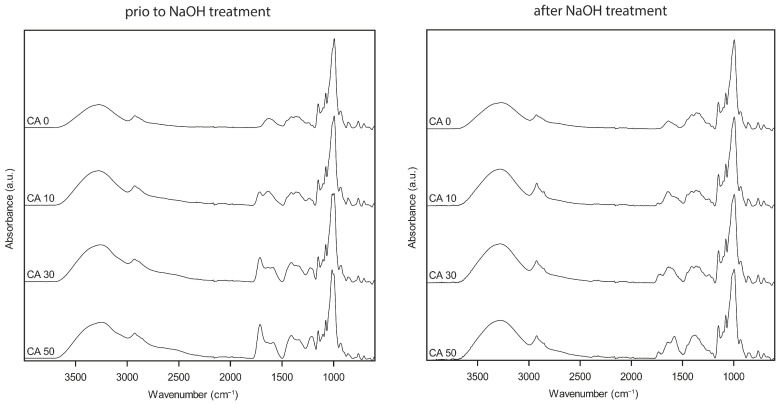
ATR-FTIR spectra in the range of 4000 to 600 cm^−1^ of potato peel-based films containing (top to bottom) 0%, 10%, 30%, or 50% (*w*/*w* potato peel) citric acid (CA) prior to and after immersion into 0.1 M NaOH.

ATR-FTIR spectra of potato peel-based films containing 10–50% CA prior to and after NaOH treatment are shown in Figure 2.

The IR spectra of potato peel-based films exhibit characteristics similar to those of starch. The broad peak around 3325 cm^−1^ and the peaks around 2920 cm^−1^, 1642 cm^−1^, and 925 cm^−1^ correspond to –OH stretching, –CH stretching, H–O–H bending of water molecules, and 1,4 glycosidic linkage vibration, respectively [41]. The differences in spectra intensity in Figure 2, particularly for the water-related peaks, may be attributed to better contact between the diamond and the coating surface due to increased water content and, thus, flexibility. Before NaOH treatment, the addition of CA (10–50%) led to the emergence of an additional peak at 1714 cm^−1^, whose intensity increased with increasing CA concentration, as compared to the peak at 1634 cm^−1^. The peak around 1714 cm^−1^ could be attributed to both carboxyl and ester carbonyl bands [20,42,43]. Therefore, no differentiation between free CA and di-esterified (cross-linked) CA could be made based on this peak.

According to Yang [36], carbonyl bands shifted from 1725 cm^−1^ to 1576 cm^−1^ after NaOH immersion of cross-linked cellulose, resulting in a decrease in the former and an increase in the latter peak, which was attributed to the carbonyl of the carboxylate anion. This allows for a differentiation between carboxylic and ester groups of free and esterified/cross-linked CA. After NaOH immersion, the peak at 1743 cm^−1^ shifted to 1732 cm^−1^ and was clearly reduced in size for all samples (Figure 2). However, only potato peel-based films containing 50% CA showed a distinguishable peak at 1578 cm^−1^, indicating the presence of a distinguishable amount of free CA. In films containing 10–30% CA, the peak at 1732 cm^−1^ was not distinguishable, but there might be a smaller one overlapping with the broader peak at 1646 cm^−1^. The different carbonyl band intensities, as well as the intensity ratios, are shown in Table 1.

The carbonyl band intensity ratio (here, 1732/1578 cm^−1^) can be used to compare differences in the degree of esterification/cross-linking [18,36]. Comparing the difference in peak intensity ratio between potato peel-based films containing 10–50% CA (Table 1), a quantitative trend in the degree of esterification and cross-linking can be derived from the ATR-FTIR analysis: CA 30 > CA 10 ≥ CA 50. An increase in CA content from 10% to 30% increased the degree of esterification/cross-linking. However, a further increase to 50% CA resulted in a decrease in the degree of esterification/cross-linking, suggesting that saturation of the cross-linking sites may have been reached at CA concentrations around 30%. Differences in the degree of esterification and cross-linking might not only be attributed to the amount of CA present but also to the pH of the film-forming suspensions, which was approximately 3.6. (CA 10), 3.1 (CA 30), and 2.3 (CA 50), respectively (Section 2.3). The decrease in the degree of esterification and cross-linking at higher CA concentrations may also be influenced by the lowering of the pH below 3, which, according to Olssen et al. [44], does not promote cross-link formation. Furthermore, the authors reported an increase in cross-linking with increasing pH [44]. However, according to Gerezgiher and Szabó [45], the highest esterification degree of non-cured CA cross-linked thermoplastic starch films was achieved when samples were processed at pH 3 without the use of pH adjustment, compared to samples that were adjusted and processed at pH 6 and pH 11 [45].

To evaluate the influence of pH changes in our samples, we adjusted the pH of potato peel-based films containing 30% CA from 2.8 ± 0.2 to pH levels of 2, 3, 4, 5, and 6 using HCl and NaOH, respectively. The ATR-FTIR spectra of pH-adjusted potato peel-based films (Figure S1) showed a gradual shift in the 1732 cm^−1^ band to lower wavenumbers as the pH increased. However, compared to the CA 30 control films, the carbonyl band intensity ratio decreased in all CA 30 pH-adjusted films after immersion in 0.1M NaOH for 2 min.

We assume that pH adjustment through the addition of HCl or NaOH does not positively influence the esterification/cross-linking of potato peel-based films. Moreover, higher intensities of the 1732 cm^−1^ band were observed at lower pH, resulting in increased carbonyl band intensity ratios. This suggests that the lowering of the film-forming suspension’s pH (from 3.6 to 2.3) with increasing CA concentration (10–50%) contributed to the rise in the carbonyl band intensity ratio. However, as the highest carbonyl band intensity ratio was observed in films containing 30% CA rather than 50% CA, the results support our hypothesis that saturation of cross-linking sites may occur at CA concentrations around 30%. Further increases in CA concentration likely led to a reduction in the degree of esterification/cross-linking.

### 3.2. Moisture Adsorption Isotherms

**Figure 3:**
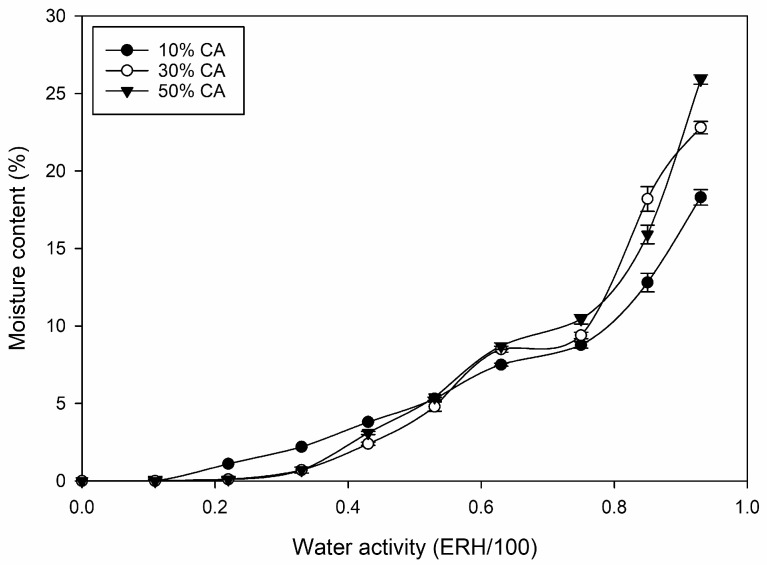
Moisture adsorption isotherms of potato peel-based films containing 10–50% (*w*/*w* potato peel) citric acid as a function of 0.0–0.93 a_w._

Moisture adsorption isotherms of potato peel-based films containing 10–50% CA showed sigmoidal shapes, which varied amongst different concentrations of CA (Figure 3). Up to a RH of 43%, all films were very brittle, with a moisture content of <4%. At 53% RH, all films had similar moisture contents of approximately 5%, but only films containing 50% CA were flexible (Section 3.3). Films containing 10% or 30% CA exhibited flexibility only at RH levels of ≥63%.

The nearly linear moisture adsorption isotherms of films containing 10% CA in the range of 0.1–0.75 a_w_ resembled those of non-plasticized starch films, confirming the negligible plasticizing effect of the low CA content. Films containing 30% and 50% CA showed decreased water sorption capacity at low a_w_ but high water sorption capacity at high a_w_, suggesting that free CA acted as a plasticizer within the potato peel matrix [46]. However, compared to other plasticizers, the overall moisture content of films containing 10–50% CA was significantly (*p* < 0.05) lower [47]. Furthermore, with increasing CA content, the moisture content of the films did not increase at the same RH, which has been reported for other plasticizers within starch-based films. This indicates that the functional sites of free CA (carboxyl groups) within the film matrix did not attract/bind water via hydrogen bonding. This finding also aligns with the overall low plasticizing ability of CA compared to other plasticizers, such as glycerol and poly-glycerol [4,5,48].

### 3.3. Tensile Properties

**Figure 4:**
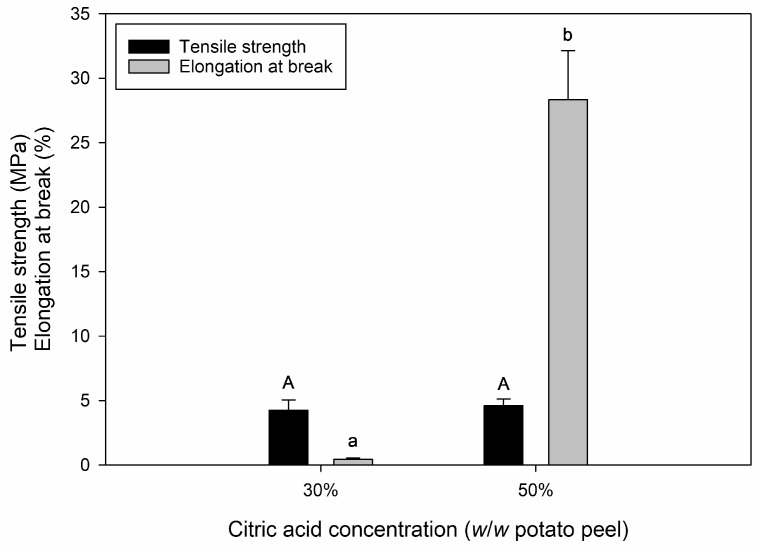
Tensile properties of potato peel-based cast films containing 30% or 50% (*w*/*w* potato peel) citric acid. The average thicknesses of the cast films containing 30% or 50% (*w*/*w* potato peel) citric acid were 131 ± 8 and 132 ± 9 µm, respectively. Different letters describe significant differences (*p* < 0.05) between measured values.

To investigate the mechanical properties of the cast films containing different amounts of CA, tensile strength and elongation at break were determined. Films containing 10% CA were too brittle and could, therefore, not be measured. The tensile properties of films containing 30% and 50% CA are shown in Figure 4.

An increase in CA concentration did not result in a significant change in tensile strength. However, with increasing CA content, elongation at break increased significantly (*p* < 0.05). The increased flexibility of the films with higher CA content indicates that CA acts as a plasticizer. It is well known that an increase in plasticizer content usually increases the flexibility of bio-based films and decreases their strength [49]. According to the results, the function of CA as a plasticizer is relatively limited compared to other plasticizers, as a concentration of 30% did not result in film plasticization and, therefore, very brittle films. Based on the results from ATR-FTIR analysis, films containing 30% CA exhibited the highest degree of cross-linking/esterification and, consequently, a low amount of free CA, which could act as a plasticizer within the films (Section 3.1). However, the moisture adsorption isotherms of potato peel-based films revealed that at approximately 50% RH, all films had a similar moisture content of around 5% (Section 3.2). This indicates that at approximately 50% RH, the increased flexibility with higher CA content was solely attributed to the plasticization effect (increased molecular spacing) of the free CA itself rather than an increase in moisture content from enhanced water retention, functioning as a plasticizer.

The drop in pH from 3.1 to 2.3 in films containing 30% and 50% CA, respectively, may contribute to the increase in film flexibility through induced hydrolysis. The pH-adjusted potato peel-based films containing 30% CA are shown in Figure S2. A decrease in pH from pH 3 to pH 2 significantly (*p* < 0.05) increased both tensile strength and elongation at break. However, since tensile strength did not increase in films containing 50% CA and the elongation at break of films containing 50% CA was more than five times higher than that of pH 2-adjusted films containing 30% CA, the influence of pH on the tensile properties only plays an inferior role. Instead, the changes in tensile properties were attributed primarily to the increase in CA concentration, leading to higher levels of free CA, which acted as a plasticizer.

### 3.4. Light Transmittance

**Figure 5:**
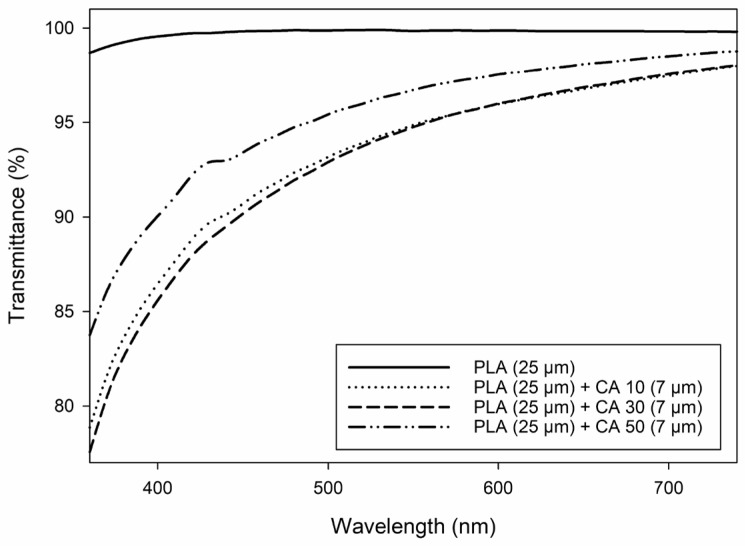
Light transmittance of PLA (25 µm) and potato peel-based coatings (7 ± 1 µm) containing 10–50% (*w*/*w* potato peel) citric acid (CA) as a function of the wavelength (right).

Coating of PLA sheets decreased light transmittance in the range of 360–740 nm, compared to neat PLA sheets (Figure 5). Overall, light transmittance decreased with decreasing wavelength, which was also previously reported for potato peel-based films plasticized with glycerol [5].

Potato peel-based coatings containing 50% CA showed significantly (*p* < 0.05) higher transmittance values in the range of 360–740 nm than coatings containing 10–30% CA. According to the results obtained from ATR-FTIR and tensile properties, films containing 50% CA were more flexible based on increased intermolecular spacing (Section 3.1 and Section 3.3), which could have also resulted in increased light transmittance values [50]. In contrast, coatings containing 10% and 30% CA exhibited a denser intermolecular network due to the small amount of free CA, acting as a plasticizer, which hindered light transmittance. The lowest light transmission was observed for coatings containing 30% CA, which may be attributed to the higher degree of cross-linking in these coatings (di-esterified CA), resulting in a denser film structure and, therefore, hindering light transmittance to a greater extent [51].

### 3.5. Oxygen, Carbon Dioxide, and Water Vapor Transmission Rate

**Figure 6:**
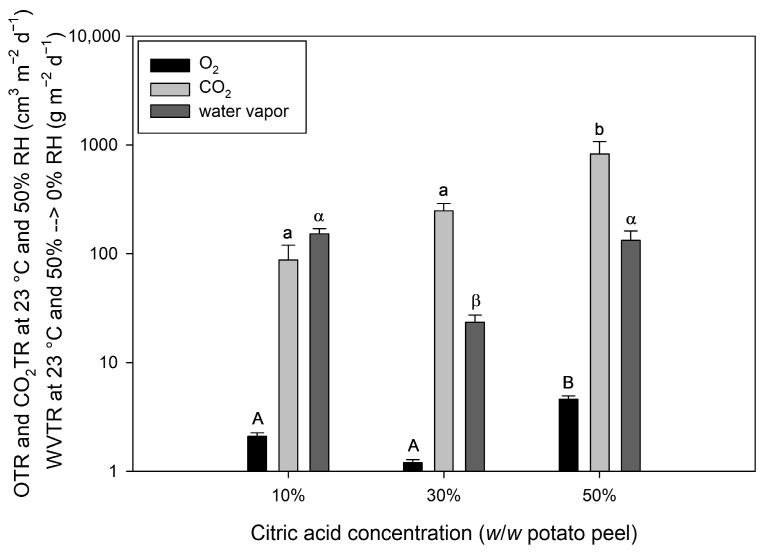
Oxygen- (OTR), carbon dioxide- (CO_2_TR), and water vapor transmission rate (WVTR) of potato peel-based coatings (7 ± 1 µm) as a function of citric acid concentration. Different letters describe significant differences (*p* < 0.05) between measured values.

Figure 6 shows the oxygen- (OTR), carbon dioxide- (CO_2_TR), and water vapor transmission rate (WVTR) of potato peel-based coatings (7 µm) as a function of CA concentration. While CO_2_TR increased with increasing CA content, the OTR and WVTR showed a non-linear correlation with minimum values observed at 30% CA content (0.08 cm^3^ m^−2^ d^−1^ bar^−1^ and 1.6 g m^−2^ d^−1^, respectively). The minimum permeability at 30% CA content was in alignment with the results obtained from ATR-FTIR measurements (Section 3.1), which showed that the addition of 30% CA led to the formation of more esters/cross-links compared to 10% and 50% CA. Moreover, the increased permeability of coatings containing 50% CA aligns with its enhanced plasticizing effect, which correlates with the films’ tensile properties (Section 3.3). Overall, the OTR of potato peel-based coatings containing 10–50% CA is comparable to those of EVOH, containing different ethylene contents [52].

Considering the influence of pH (2–6) (Figure S3), no significant differences in OTR, CO_2_TR, and WVTR were observed for potato peel-based coatings containing 30% CA derived from pH 3 to 5-adapted film-forming suspensions. An increase in the WVTR for pH 6-adapted coatings is likely attributed to the observed crystallization of CA within and on the surface of the coatings, resulting in increased water absorption in these hygroscopic regions. However, a pH adjustment to pH 2 resulted in significantly increased OTR, CO_2_TR, and WVTR values. The increase in permeability upon lowering the pH of the formulation using HCl could be attributed to the induced hydrolysis of polymer chains. Hydrolysis exposes more functional groups, resulting in additional non-covalent bonds, such as hydrogen bonds. Consequently, increased intermolecular spacing and free volume likely contributed to the rise in permeability, which aligns with the increased film flexibility (Section 3.3). Furthermore, the increased film permeability may also be attributed to enhanced hydrolysis caused by the addition of 50% CA and the resulting pH reduction to 2.3.

**Figure 7:**
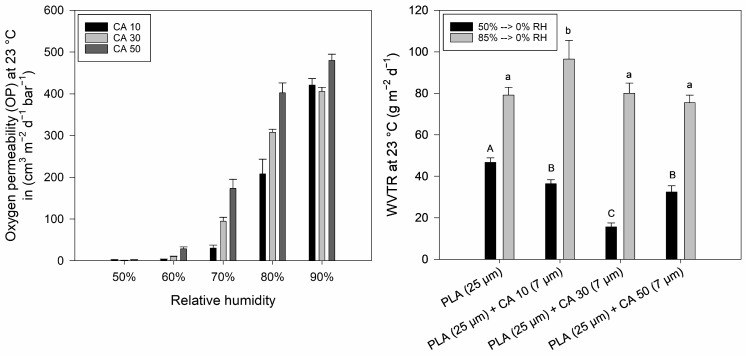
Oxygen permeability of potato peel-based coatings (7 ± 1 µm) containing 10–50% (*w*/*w* potato peel) citric acid (CA) as a function of relative humidity (**left**) and water vapor transmission rate (WVTR) of PLA and PLA–potato peel bi-layers containing 10–50% (*w*/*w* potato peel) CA (**right**). Different letters describe significant differences (*p* < 0.05) between measured values.

To investigate the influence of RH on potato peel-based coating permeability, RH dependent OP and WVTR measurements were performed (Figure 7). In the range of 50–80% RH, OP increased exponentially with rising RH, illustrating the non-linear relationship between the permeability of hydrophilic biopolymers and RH. Between 60% and 80% RH, OP correlates with CA concentration and moisture adsorption isotherms, which showed increased moisture content values for films containing higher concentrations of CA at an RH of ≥63% (Section 3.2).

The WVTRs of PLA and PLA–potato peel bi-layers increased significantly (*p* < 0.05) when measured at 85% RH compared to 50% RH. At 85% RH, the 7 µm thick potato peel-based coating containing 30–50% CA did not significantly affect the WVTR of the PLA substrate. Only coatings with 10% CA significantly (*p* < 0.05) increased the measured WVTR. However, at 50% RH, all coatings significantly (*p* < 0.05) decreased the WVTR of the bi-layers when compared to the PLA substrate. The low WVTRs of potato peel-based coatings containing 10–50% CA at 50% RH might be correlated to their low moisture content (Section 3.2) and overall dense structure due to the formation of a significant amount of esters/cross-links (Section 3.1 and Section 3.3). Thus, the WVTRs of potato peel-based coatings containing 10–50% CA at 50% RH are remarkably lower than that of potato peel-based films using other plasticizers [4,5,53].

## 4. Conclusions

In this study, the impact of CA at concentrations of 10–50% (*w*/*w* potato peel) on the structure and physicochemical properties of potato peel-based films and coatings were characterized using ATR-FTIR, moisture adsorption isotherms, tensile testing, light transmittance measurements, and humidity-dependent barrier measurements against oxygen, carbon dioxide, and water vapor. The findings indicate that increasing CA concentrations up to 30% enhanced esterification/cross-linking, while higher concentrations (>30%) increased the amount of free CA, contributing to its plasticizing function. Unlike other plasticizers, CA acted as a relatively non-hygroscopic plasticizer, improving film flexibility without significantly increasing moisture content. Consequently, film properties were primarily influenced by the difference in intermolecular spacing from cross-links of esterified CA or hydrogen bonds of free CA rather than water retention in the film matrix. This resulted in brittle films at low relative humidity (<63%) and overall low gas and vapor permeability. Achieving flexible cast films required relatively high CA concentrations (>30%). Due to the brittleness of potato peel-based films/coatings containing 10–30% (*w*/*w* potato peel) CA, especially at low relative humidity, the addition of an additional plasticizer, such as glycerol, may be beneficial to increase flexibility. Therefore, the effect of CA as a cross-linking agent should be investigated in combination with other plasticizers in order to further optimize the films/coatings with regard to their mechanical and barrier properties. However, the coating application of suspensions containing relatively low amounts of CA (≥10%) was still feasible without cracking the coating when stored at ambient conditions (23 °C, 50% RH). Potato peel-based suspensions varied in pH (2.3–3.6) depending on the CA concentration (10–50%). The pH adjustment of CA 30 films (pH 2–6) influenced ATR-FTIR measurements and the physicochemical properties of the films/coatings, suggesting the need for further detailed investigations into the role of pH. Additionally, future studies should examine the molecular interactions between CA and the various components in potato peel that serve as cross-linking sites under different pH conditions.

In conclusion, potato peel-based coatings containing 10–50% (*w*/*w* potato peel) CA exhibited excellent barrier properties against water vapor at 23 °C and 50% RH, along with oxygen barrier properties comparable to EVOH, despite the absence of extensive industrial purification or fractionation of the potato peel. These findings highlight the significant potential of potato peel-based films and coatings while also emphasizing the need for further research to advance the use of this by-product for various packaging applications. For instance, the presence of undissolved particles in potato peel suspensions limits the minimum achievable coating thickness that could be applied. To achieve thinner coatings comparable to those of EVOH, further reduction of the potato peel powder particle size and/or the use of alternative solvents should be considered. Furthermore, the low dry matter content of the potato peel suspension necessitates extended drying times to remove excess water, potentially requiring longer drying ovens or reducing maximum production speed. Nevertheless, alternative coating techniques, such as reverse (micro) gravure, knife-over-edge, or slot-die coating, may offer viable solutions for scaling up to a roll-to-roll coating process [54] and should be explored in future research.

## Figures and Tables

**Table 1:** Carbonyl band intensities at 1732 cm^−1^, 1578 cm^−1^, and their ratios obtained from threefold ATR-FTIR measurements of potato peel-based films containing 10–50% (*w*/*w* potato peel) citric acid (CA) after NaOH immersion.

Carbonyl Band (cm^−1^)	Absorbance (a.u)		
	CA 10	CA 30	CA 50
1732	0.011/0.009/0.012	0.025/0.027/0.030	0.019/0.031/0.016
1578	0.049/0.020/0.034	0.025/0.029/0.060	0.064/0.091/0.057
1732/1578	0.33 ± 0.10	0.81 ± 0.26	0.30 ± 0.03

## Data Availability

The original contributions presented in the study are included in the article; further inquiries can be directed to the corresponding authors.

## Supplementary Materials

The following supporting information can be downloaded at https://www.mdpi.com/article/10.3390/polym16243506/s1, Figure S1: ATR-FTIR spectra in the range of 4000 to 600 cm^−1^ of potato peel-based films containing 30% CA (*w*/*w* potato peel) as a function of pH (film-forming suspension) prior to and after immersion into 0.1 M NaOH; Figure S2: Tensile properties of potato peel-based cast films containing 30% (*w*/*w* potato peel) citric acid as a function of pH (film-forming suspension). Average thicknesses of the cast films (pH 2, 3, 4, 5, and 6) were 123 ± 9, 124 ± 10, 141 ± 8, 147 ± 11, and 150 ± 5 µm, respectively. Different letters describe significant differences (*p* < 0.05) between measured values.; Figure S3: Oxygen- (OTR), carbon dioxide- (CO_2_TR), and water vapor transmission rate (WVTR) of potato peel-based coatings (7 ± 1 µm) containing 30% CA (*w*/*w* potato peel) as a function of pH (film-forming suspension). Different letters describe significant differences (*p* < 0.05) between measured values.

## References

[1] Ebrahimian F., Denayer J.F.M., Karimi K. (2022). Potato peel waste biorefinery for the sustainable production of biofuels, bioplastics, and biosorbents. Bioresour. Technol..

[2] Kang H.J., Won M.Y., Lee S.J., Min S.C. (2015). Plasticization and moisture sensitivity of potato peel-based biopolymer films. Food Sci. Biotechnol..

[3] Borah P.P., Das P., Badwaik L.S. (2017). Ultrasound treated potato peel and sweet lime pomace based biopolymer film development. Ultrason. Sonochem..

[4] Merino D., Paul U.C., Athanassiou A. (2021). Bio-based plastic films prepared from potato peels using mild acid hydrolysis followed by plasticization with a polyglycerol. Food Packag. Shelf Life.

[5] Miller K., Silcher C., Lindner M., Schmid M. (2021). Effects of glycerol and sorbitol on optical, mechanical, and gas barrier properties of potato peel-based films. Packag. Technol. Sci..

[6] Jahangiri F., Mohanty A.K., Misra M. (2024). Sustainable biodegradable coatings for food packaging: Challenges and opportunities. Green Chem..

[7] Juikar S.K., Warkar S.G. (2023). Biopolymers for packaging applications: An overview. Packag. Technol. Sci..

[8] Miller K., Reichert C.L., Schmid M., Loeffler M. (2022). Physical, Chemical and Biochemical Modification Approaches of Potato (Peel) Constituents for Bio-Based Food Packaging Concepts: A Review. Foods.

[9] Yao S., Wang B.-J., Weng Y.-M. (2022). Preparation and characterization of mung bean starch edible films using citric acid as cross-linking agent. Food Packag. Shelf Life.

[10] Seligra P.G., Medina Jaramillo C., Famá L., Goyanes S. (2016). Biodegradable and non-retrogradable eco-films based on starch-glycerol with citric acid as crosslinking agent. Carbohydr. Polym..

[11] Azeredo H.M., Waldron K.W. (2016). Crosslinking in polysaccharide and protein films and coatings for food contact—A review. Trends Food Sci..

[12] Zhang W., Roy S., Assadpour E., Cong X., Jafari S.M. (2023). Cross-linked biopolymeric films by citric acid for food packaging and preservation. Adv. Colloid Interface Sci..

[13] Castro J.M., Montalbán M.G., Martínez-Pérez N., Domene-López D., Pérez J.M., Arrabal-Campos F.M., Fernández I., Martín-Gullón I., García-Quesada J.C. (2023). Thermoplastic starch/polyvinyl alcohol blends modification by citric acid-glycerol polyesters. Int. J. Biol. Macromol..

[14] Dudeja I., Mankoo R.K., Singh A., Kaur J. (2023). Citric acid: An ecofriendly cross-linker for the production of functional biopolymeric materials. Sustain. Chem. Pharm..

[15] Salihu R., Razak S.I.A., Zawawi N.A., Kadir M.R.A., Ismail N.I., Jusoh N., Mohamad M.R., Nayan N.H.M. (2021). Citric acid: A green cross-linker of biomaterials for biomedical applications. Eur. Polym. J..

[16] Jayachandran B., Parvin T.N., Alam M.M., Chanda K., MM B. (2022). Insights on Chemical Crosslinking Strategies for Proteins. Molecules.

[17] Li T., Hu J., Tian R., Wang K., Li J., Qayum A., Bilawal A., Gantumur M.-A., Jiang Z., Hou J. (2021). Citric acid promotes disulfide bond formation of whey protein isolate in non-acidic aqueous system. Food Chem..

[18] de Cuadro P., Belt T., Kontturi K.S., Reza M., Kontturi E., Vuorinen T., Hughes M. (2015). Cross-linking of cellulose and poly(ethylene glycol) with citric acid. React. Funct. Polym..

[19] Nataraj D., Sakkara S., HN M., Reddy N. (2018). Properties and applications of citric acid crosslinked banana fibre-wheat gluten films. Ind. Crops.

[20] Reddy N., Yang Y. (2010). Citric acid cross-linking of starch films. Food Chem..

[21] Montilla-Buitrago C.E., Gómez-López R.A., Solanilla-Duque J.F., Serna-Cock L., Villada-Castillo H.S. (2021). Effect of Plasticizers on Properties, Retrogradation, and Processing of Extrusion-Obtained Thermoplastic Starch: A Review. Starch-Stärke.

[22] Menzel C. (2020). Improvement of starch films for food packaging through a three-principle approach: Antioxidants, cross-linking and reinforcement. Carbohydr. Polym..

[23] Gebresas G.A., Szabó T., Marossy K. (2023). A comparative study of carboxylic acids on the cross-linking potential of corn starch films. J. Mol. Struct..

[24] Chen W.C., Judah S.N.M.S.M., Ghazali S.K., Munthoub D.I., Alias H., Mohamad Z., Majid R.A. (2021). The Effects of Citric Acid on Thermal and Mechanical Properties of Crosslinked Starch Film. Chem. Eng. Trans..

[25] Wu H., Lei Y., Lu J., Zhu R., Xiao D., Jiao C., Xia R., Zhang Z., Shen G., Liu Y. (2019). Effect of citric acid induced crosslinking on the structure and properties of potato starch/chitosan composite films. Food Hydrocoll..

[26] Azeredo H.M., Kontou-Vrettou C., Moates G.K., Wellner N., Cross K., Pereira P.H., Waldron K.W. (2015). Wheat straw hemicellulose films as affected by citric acid. Food Hydrocoll..

[27] Menzel C., Olsson E., Plivelic T.S., Andersson R., Johansson C., Kuktaite R., Järnström L., Koch K. (2013). Molecular structure of citric acid cross-linked starch films. Carbohydr. Polym..

[28] Olsson E., Hedenqvist M.S., Johansson C., Järnström L. (2013). Influence of citric acid and curing on moisture sorption, diffusion and permeability of starch films. Carbohydr. Polym..

[29] Sakkara S., Venkatesh K., Reddy R., Nagananda G.S., Meghwal M., Patil J.H., Reddy N. (2020). Characterization of crosslinked Macrotyloma uniflorum (Horsegram) protein films for packaging and medical applications. Polym. Test..

[30] Sharmin N., Sone I., Walsh J.L., Sivertsvik M., Fernández E.N. (2021). Effect of citric acid and plasma activated water on the functional properties of sodium alginate for potential food packaging applications. Food Packag. Shelf Life.

[31] Wang S., Ren J., Li W., Sun R., Liu S. (2014). Properties of polyvinyl alcohol/xylan composite films with citric acid. Carbohydr. Polym..

[32] Uranga J., Puertas A.I., Etxabide A., Dueñas M.T., Guerrero P., de la Caba K. (2019). Citric acid-incorporated fish gelatin/chitosan composite films. Food Hydrocoll..

[33] Wen L., Liang Y., Lin B., Xie D., Zheng Z., Xu C., Lin Z. (2021). Design of multifunctional food packaging films based on carboxymethyl chitosan/polyvinyl alcohol crosslinked network by using citric acid as crosslinker. Polymer.

[34] Bisht B., Gururani P., Aman J., Vlaskin M.S., Anna I.K., Irina A.A., Joshi S., Kumar S., Kumar V. (2023). A review on holistic approaches for fruits and vegetables biowastes valorization. Mater. Today Proc..

[35] Miller K., Reichert C.L., Loeffler M., Schmid M. (2024). Effect of Particle Size on the Physical Properties of PLA/Potato Peel Composites. Compounds.

[36] Yang C.Q. (1991). Characterizing Ester Crosslinkages in Cotton Cellulose with FT-IR Photoacoustic Spectroscopy. Text. Res. J..

[37] (2019). Kunststoffe—Bestimmung der Zugeigenschaften—Teil 3: Prüfbedingungen für Folien und Tafeln.

[38] (1998). Bestimmung der Gasdurchlässigkeit: Teil 3: Sauerstoffspezifisches Trägergas-Verfahren zur Messung.

[39] (2003). Plastics—Film and Sheeting—Determination of Gas-Transmission Rate—Part 2: Equal-Pressure Method.

[40] (2001). Bestimmung der Wasserdampfdurchlässigkeit: Teil 1: Gravimetrisches Verfahren.

[41] Semlali Aouragh Hassani F.-Z., Salim M.H., Kassab Z., Sehaqui H., Ablouh E.-H., Bouhfid R., Qaiss A.E.K., El Achaby M. (2022). Crosslinked starch-coated cellulosic papers as alternative food-packaging materials. RSC Adv..

[42] Shi R., Zhang Z., Liu Q., Han Y., Zhang L., Chen D., Tian W. (2007). Characterization of citric acid/glycerol co-plasticized thermoplastic starch prepared by melt blending. Carbohydr. Polym..

[43] Wilpiszewska K., Antosik A.K., Zdanowicz M. (2019). The Effect of Citric Acid on Physicochemical Properties of Hydrophilic Carboxymethyl Starch-Based Films. J. Polym. Environ..

[44] Olsson E., Menzel C., Johansson C., Andersson R., Koch K., Järnström L. (2013). The effect of pH on hydrolysis, cross-linking and barrier properties of starch barriers containing citric acid. Carbohydr. Polym..

[45] Gerezgiher A.G., Szabó T. (2022). Crosslinking of Starch Using Citric Acid. J. Phys. Conf. Ser..

[46] Jiménez A., Fabra M.J., Talens P., Chiralt A. (2013). Phase transitions in starch based films containing fatty acids. Effect on water sorption and mechanical behaviour. Food Hydrocoll..

[47] Xu X., Wang Y., Huang F.-T., Du K., Nowadnick E.A., Cheong S.-W. (2020). Highly tunable ferroelectricity in hybrid improper ferroelectric Sr_3_Sn_2_O_7_. Adv. Funct. Mater..

[48] Rommi K., Rahikainen J., Vartiainen J., Holopainen U., Lahtinen P., Honkapää K., Lantto R. (2016). Potato peeling costreams as raw materials for biopolymer film preparation. J. Appl. Polym. Sci..

[49] Laohakunjit N., Noomhorm A. (2004). Effect of Plasticizers on Mechanical and Barrier Properties of Rice Starch Film. Starch-Stärke.

[50] Edhirej A., Sapuan S.M., Jawaid M., Zahari N.I. (2017). Effect of various plasticizers and concentration on the physical, thermal, mechanical, and structural properties of cassava-starch-based films. Starch-Stärke.

[51] Wang Y., Chen S., Yao Y., Wu N., Xu M., Yin Z., Zhao Y., Tu Y. (2023). Effects of citric acid crosslinking on the structure and properties of ovotransferrin and chitosan composite films. Int. J. Biol. Macromol..

[52] Maes C., Luyten W., Herremans G., Peeters R., Carleer R., Buntinx M. (2018). Recent Updates on the Barrier Properties of Ethylene Vinyl Alcohol Copolymer (EVOH): A Review. Polym. Rev..

[53] Othman S.H., Edwal S.A.M., Risyon N.P., Basha R.K., Talib R.A. (2017). Water sorption and water permeability properties of edible film made from potato peel waste. Food Sci. Technol..

[54] Park J., Shin K., Lee C. (2016). Roll-to-Roll Coating Technology and Its Applications: A Review. Int. J. Precis. Eng. Manuf..

