# Supplementary material for: Impact of Citric Acid on the Structure, Barrier, and Tensile Properties of Esterified/Cross-Linked Potato Peel-Based Films and Coatings
Source: Polymers (Basel). 2024 Dec 17;16(24):3506. doi: 10.3390/polym16243506 (PMC11679704; doi:10.3390/polym16243506)
Supplement: Supplementary file 1 [file polymers-16-03506-s001.zip › polymers-3278428-supplementary.pdf]

Supplementary Material:

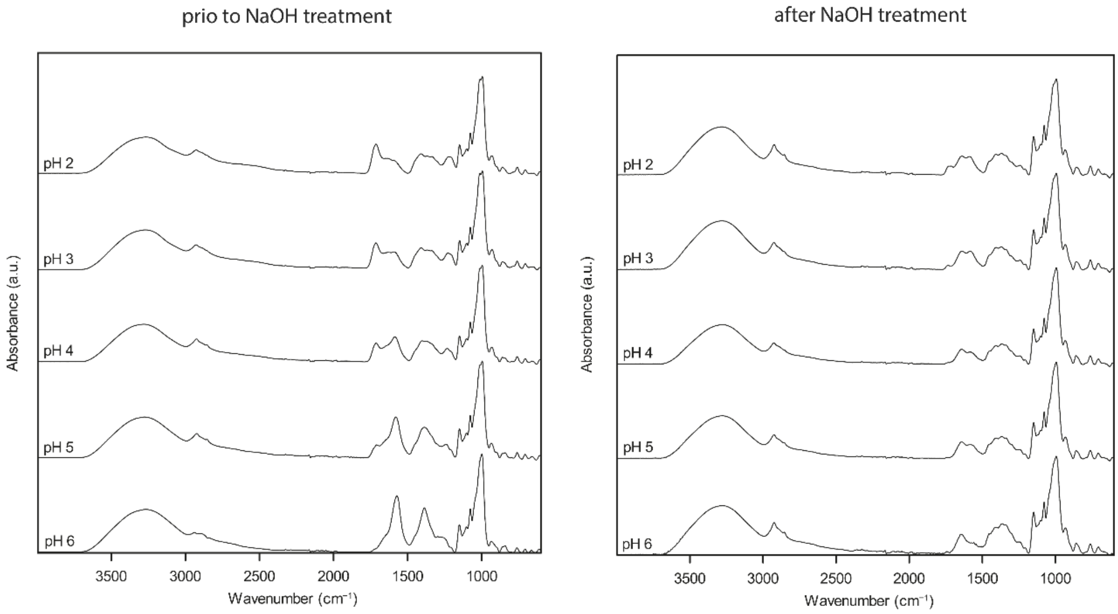

Figure S1: ATR-FTIR spectra in the range of 4000 to 600 cm^{-1} of potato peel-based films containing 30% CA (*w/w* potato peel) as a function of pH (film-forming suspension) prior and after immersion into 0.1 M NaOH.

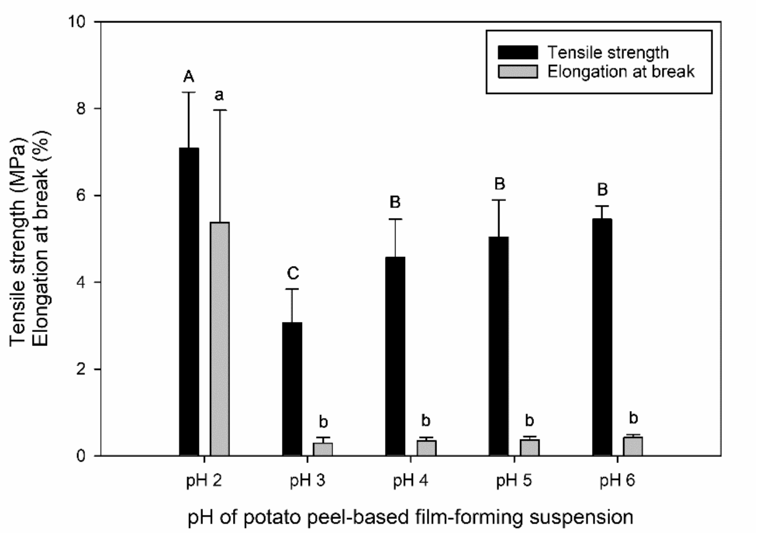

Figure S2. Tensile properties of potato peel-based cast films containing 30% (*w/w* potato peel) citric acid as a function of pH (film-forming suspension). Average thicknesses of the cast films (pH 2, 3, 4, 5, and 6) were 123 ± 9, 124 ± 10, 141 ± 8, 147 ± 11, and 150 ± 5 μm, respectively. Different letters describe significant differences ($p < 0.05$) between measured values.

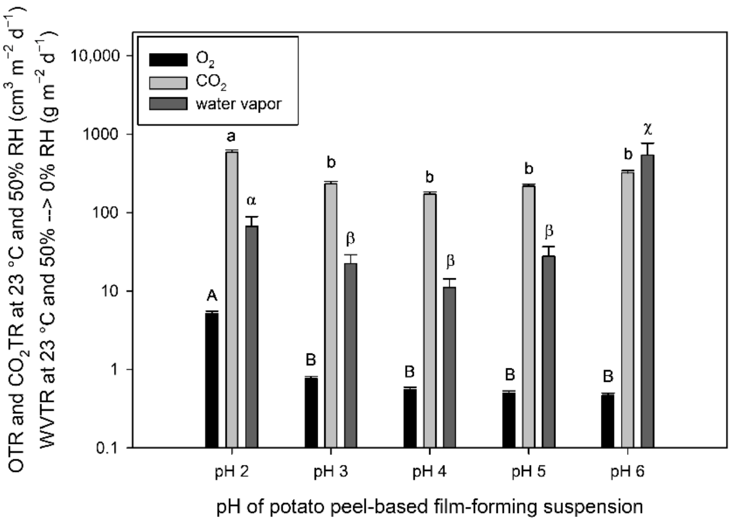

Figure S3: Oxygen- (OTR), carbon dioxide- (CO_2TR), and water vapor transmission rate (WVTR) of potato peel-based coatings (7 ± 1 μm) containing 30% CA (*w*/*w* potato peel) as a function of pH (film-forming suspension). Different letters describe significant differences ($p < 0.05$) between measured values.
